# Supplementary material for: The interactome of intact mitochondria by cross-linking mass spectrometry provides evidence for coexisting respiratory supercomplexes
Source: Mol Cell Proteomics. 2017 Dec 8;17(2):216–32. doi: 10.1074/mcp.RA117.000470 (PMC5795388; doi:10.1074/mcp.RA117.000470)
Supplement: Supplemental Data [file supp_RA117.000470_133922_0_supp_23978_fzffwf.zip › spectra_annotation/mito_DR_spectra_annotation/423-1-10-1-6-1.pdf]

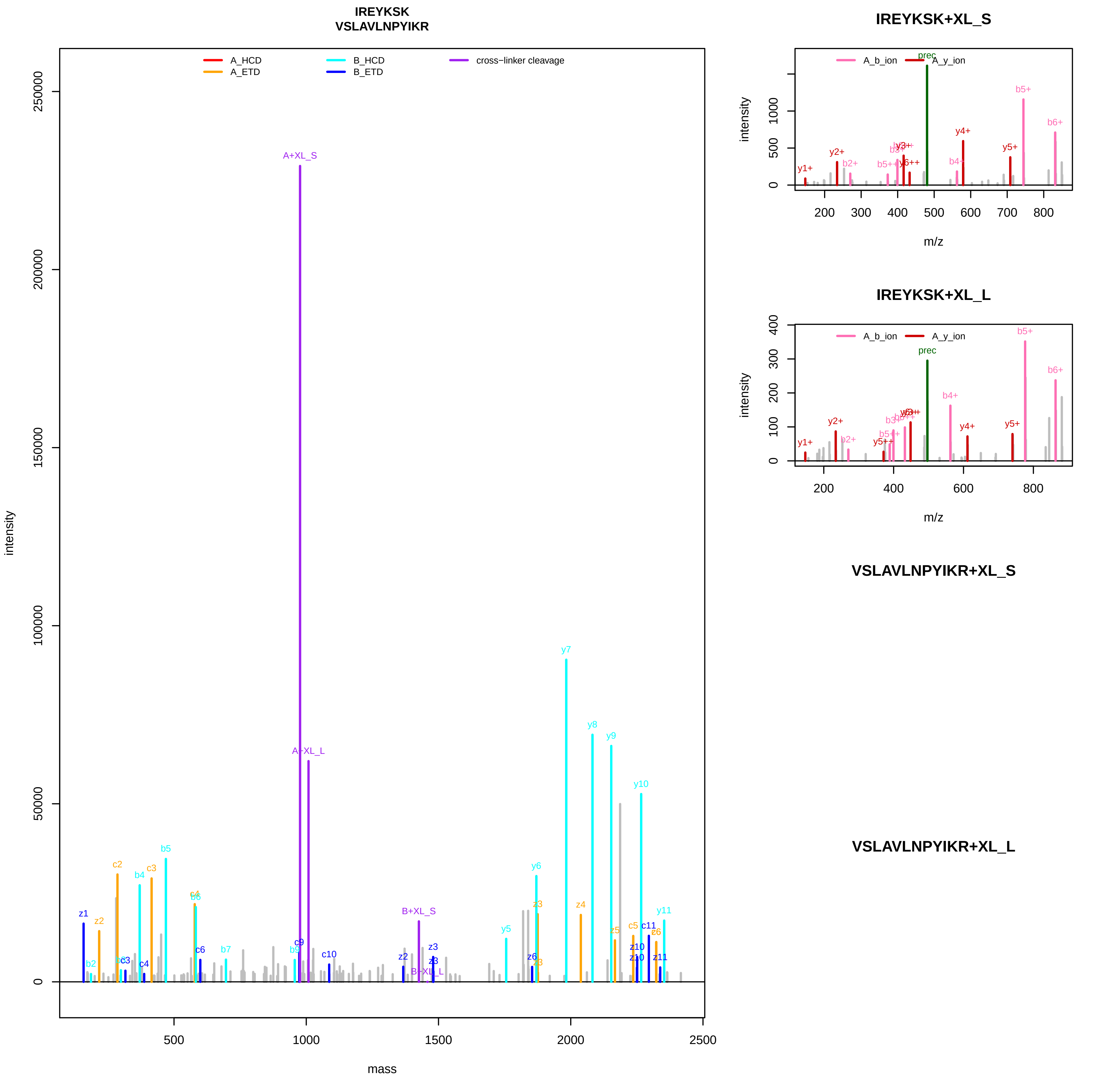
IREYKSK
VSLAVLNPYIKR
A_HCD
A_ETD
B_HCD
B_ETD
cross−linker cleavage
A+XL_S
A+XL_L
B+XL_S
B+XL_L
intensity
mass
0
50000
100000
150000
200000
250000
500
1000
1500
2000
2500
z1 b2 z2 c2 b3 c3 b4 c4 c3 b5 c4 b6 c6 b7 b9 c9 c10 z2 z3 z3 z6 y5 y6 z3 z3 y7 z4 y8 y9 z5 c5 y10 z10 z10 c11 z6 z11 y11
IREYKSK+XL_S
IREYKSK+XL_L
A_b_ion
A_y_ion
prec
y1+ y2+ b2+ b3+ y3+ b5++ y6++ y4+ b4+ y5+ b5+ b6+
intensity
m/z
0
500
1000
100
200
300
400
200
300
400
500
600
700
800
VSLAVLNPYIKR+XL_S
VSLAVLNPYIKR+XL_L
